# Bone Tissue Response to Different Grown Crystal Batches of Octacalcium Phosphate in Rat Long Bone Intramedullary Canal Area

**DOI:** 10.3390/ijms22189770

**Published:** 2021-09-09

**Authors:** Yukari Shiwaku, Ryo Hamai, Shinichi Sato, Susumu Sakai, Kaori Tsuchiya, Kazuyoshi Baba, Tetsu Takahashi, Osamu Suzuki

**Affiliations:** 1 Division of Craniofacial Function Engineering, Tohoku University Graduate School of Dentistry, Sendai 980-8575, Japan; yukari.shiwaku.a8@tohoku.ac.jp (Y.S.); ryo.hamai.a3@tohoku.ac.jp (R.H.); shinichi.sato.c4@tohoku.ac.jp (S.S.); susumu.sakai.p8@dc.tohoku.ac.jp (S.S.); kaori.tsuchiya.b6@tohoku.ac.jp (K.T.); kazuyoshi.baba.e3@tohoku.ac.jp (K.B.); 2 Liaison Center for Innovative Dentistry, Tohoku University Graduate School of Dentistry, Sendai 980-8575, Japan; 3 Division of Oral and Maxillofacial Surgery, Tohoku University Graduate School of Dentistry, Sendai 980-8575, Japan; tetsu@dent.tohoku.ac.jp; 4 Department of Orthopaedic Surgery, Tohoku University Graduate School of Medicine, Sendai 980-8575, Japan

**Keywords:** octacalcium phosphate, crystal microstructure, bone formation, solubility equilibrium, osteoclast-like cells

## Abstract

The microstructure of biomaterials influences the cellular and biological responses in the bone. Octacalcium phosphate (OCP) exhibits higher biodegradability and osteoconductivity than hydroxyapatite (HA) during the conversion process from OCP to HA. However, the effect of the microstructure of OCP crystals on long tubular bones has not been clarified. In this study, two types of OCPs with different microstructures, fine-OCP (F-OCP) and coarse-OCP (C-OCP), were implanted in rat tibia for 4 weeks. F-OCP promoted cortical bone regeneration compared with C-OCP. The osteoclasts appearance was significantly higher in the C-OCP group than in the control group (defect only) at 1-week post-implantation. To investigate whether the solubility equilibrium depends on the different particle sizes of OCPs, Nano-OCP, which consisted of nanometer-sized OCPs, was prepared. The degree of supersaturation (DS) tended to decrease modestly in the order of C-OCP, F-OCP, and Nano-OCP with respect to HA and OCP in Tris-HCl buffer. F-OCP showed a higher phosphate ion concentration and lower calcium ion concentration after immersion in the buffer than C-OCP. The crystal structures of both OCPs tended to be converted to HA by rat abdominal implantation. These results suggest that differences in the microstructure of OCPs may affect osteoclastogenesis and result in osteoconductivity of this material in long tubular bone by altering dissolution behavior.

## 1. Introduction

Osteoconductive inorganic bone substitute materials include calcium phosphate (CaP) ceramic, such as sintered hydroxyapatite (HA), beta-tricalcium phosphate (β-TCP), biphasic calcium phosphate (BCP), octacalcium phosphate (OCP), calcium cement, glass-ceramic and bioglass. Some of the materials are widely used in orthopedic surgery and dentistry. The material characteristics of bone substitutes, such as chemical solubility [1], granule size [2], and crystal morphology [3], affect cellular function, material resorption, and osteoconductivity. Therefore, there is a need to develop biomaterials with bone regeneration ability comparable to the autogenous bone by controlling the physicochemical characteristics of CaP crystals to suit clinical applications.

The microstructure of biomaterials influences biological cellular responses. For example, an increase in the surface area and microstructure of BCP can concentrate more proteins and affect cell adhesion, proliferation, and differentiation [4,5]. The surface microstructure of β-TCP has also been reported to affect osteogenic differentiation of mesenchymal stem cells and ectopic bone formation [6]. The surface microstructure of BCP modulates osteoclastogenesis and alters ectopic bone formation [7]. Furthermore, the microstructure of bioglass (30 mol% CaO-70 mol% SiO_2_ porous bioactive glass) promotes MC3T3 cell adhesion as the β-sheet/α-helix ratio of adsorbed BSA protein increases [8].

OCP is a bioresorbable calcium phosphate that exhibits superior osteoconductive properties compared to HA and β-TCP in calvaria or long tubular bone (tibia and femur) defects in mice, rats, and rabbits [9,10]. In recent years, a composite of OCP and collagen has been used in clinical practice for bone augmentation in dental implants and cleft palate treatment. OCP has unique characteristics where it can convert to HA in vitro and in vivo. This conversion process enhances the activity of various bone-related cells. We have previously shown that OCP promotes the differentiation of osteoblasts from mesenchymal stem cells during OCP-HA conversion [11,12], enhancing bone formation. Osteoblasts are embedded in the bone matrix and undergo final differentiation into osteocytes. OCP promotes the differentiation of osteoblasts into osteocytes in vitro and in vivo [13,14]. It has also been shown that the addition of amorphous calcium phosphate (ACP) to OCP promotes bone regeneration in rat calvarial defects [15].

OCP-HA conversion affects osteogenesis, inflammation, and material resorption. OCP regulates macrophage migration by altering Ca^2+^ concentration [16] and promotes osteoclast formation via receptor activator of nuclear factor-kappa B ligand (RANKL) expression in osteoblasts [17]. Differences in the crystal phase of OCP and HA have also been shown to promote osteoblast differentiation by changing the expression of coupling factors secreted by osteoclasts [18].

Various material properties of OCP and OCP–HA conversion have been shown to affect bone regeneration in calvarial defects. For example, the larger granule diameter of OCP (500–1000 μm) promotes osteogenesis and osteoclast-like cell formation in mouse calvarial defects [2]. The crystal length of OCP affects the adhesion of mouse bone marrow-derived stromal cells and osteogenesis in mouse calvarial defects [3].

OCP also promotes bone regeneration in the long tubular bones. In rat tibia, slightly hydrolyzed OCP weakened the initial inflammatory response and accelerated bone formation compared with the original OCP [19]. OCP can be complexed with various polymers, such as gelatin and poly lactide-co-glycolide acid (PLGA). Even in the complexes, the solubility and ionic behavior of OCP change cellular activity and osteoconductivity [20,21].

It is widely known that the process of ossification in the developmental stage differs between calvaria and long tubular bones. Calvarial bone is formed by membranous ossification, and periosteum-derived cells are thought to be involved in bone regeneration [22]. In contrast, long bones are formed by calcification of cartilage through endochondral ossification, and bone marrow-derived cells are involved in regeneration [23]. However, the influence of the OCP material properties on bone regeneration with different ossification has not yet been clarified.

In this study, among the various properties of OCP, we focused on the difference in crystal size (microstructure). Two types of OCPs were fabricated in the laboratory: (1) Fine-OCP (F-OCP), an aggregate composed of small needle-shaped crystals, and (2) coarse-OCP (C-OCP), an aggregate composed of plate-like crystals well-grown toward the long axis of the crystals. In calvarial defects, F-OCP enhances the adhesion of bone marrow-derived cells and promotes bone formation [3]. However, the cellular response and bone regeneration ability of long bones are still unknown. This study evaluated the crystal structure and solubility of F-OCP and C-OCP and investigated the effect of OCP microstructure on bone regeneration ability in long tubular bone through micro-CT and histological analysis. 

## 2. Results

### 2.1. Crystal Morphology of F-OCP and C-OCP

**Figure 1:**
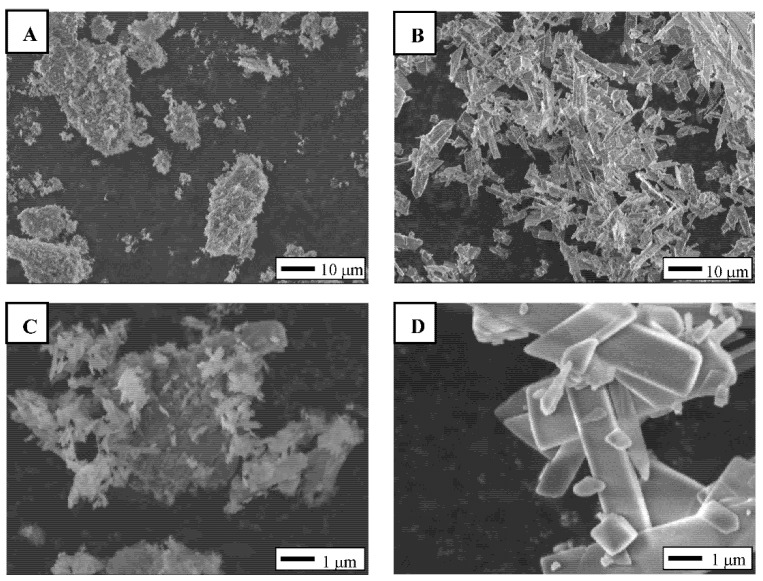
FE-SEM images of F-OCP (**A**,**C**) and C-OCP granules (**B**,**D**) at lower (**A**,**B**) and higher magnifications (**C**,**D**). Bars = 10 µm (**A**,**B**) and 1 µm (**C**,**D**).

The crystal morphologies of the prepared F-OCP and C-OCP were observed using field emission scanning electron microscopy (FE-SEM) (Figure 1). F-OCP crystals formed aggregates with spherical shapes with lengths ranging from 10 μm to 50 μm and widths ranging from 10 μm to 30 μm (Figure 1A,C). Needle-shaped crystals with approximately 100–200 nm widths and about 0.5–1.0 μm lengths were observed on F-OCP aggregates. In contrast, C-OCP formed some aggregates consisting of plate-like crystals ranged from 0.3 μm to 2.0 μm in width and 1 μm to 30 μm in length (Figure 1B,D).

### 2.2. Change of Crystal Structure of OCPs after Immersion in the Tris-HCl Buffer

**Figure 2:**
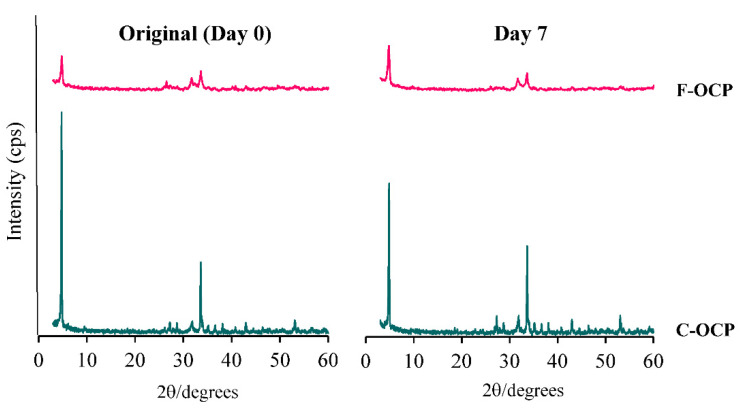
X-ray diffraction patterns of F-OCP and C-OCP at the original (day 0) and day 7 after immersion in Tris-HCl buffer.

To investigate the solubility of each crystal, F-OCP and C-OCP were immersed in 150 mM Tris-HCl buffer (pH = 7.4) for 7 days at 37 °C. X-ray diffraction (XRD) analysis of F-OCP and C-OCP, before immersion, showed diffraction peaks at 2θ = 4.8° and 33.6°, corresponding to the (100) and (700) planes, respectively, of the OCP crystals (Figure 2). In particular, the full widths at half maximum at 2θ = 4.8° and 33.6° of C-OCP (0.149, 0.148 (deg)) were smaller than those of F-OCP (0.333, 0.374 (deg)) in the original state, suggesting that C-OCP possessed higher crystallinity. 

After 7 days of immersion in the Tris-HCl buffer, the (100) peak of C-OCP decreased compared to that of the original, indicating that the hydrolysis was slightly advanced. However, in F-OCP, the peak of (100) was the same as before immersion, suggesting that hydrolysis had not progressed. No specific peaks of HA (2θ = 10.8°) were observed in either OCP on day 7 after immersion in Tris-HCl buffer. These results suggest that F-OCP and C-OCP retained the OCP crystal structure.

**Figure 3:**
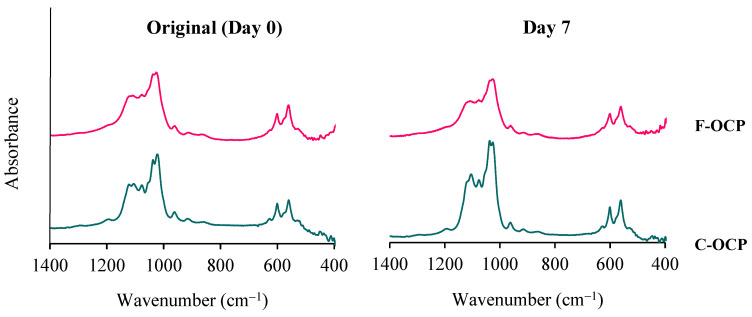
FTIR spectra of F-OCP and C-OCP at the original (day 0) and day 7 after immersion in Tris-HCl buffer.

The Fourier transform infrared spectroscopy (FTIR) analysis showed that both F-OCP and C-OCP had five characteristic peaks of OCP (Figure 3). Two sharp adsorption bands originating from ν_4_ PO_4_ at approximately 560–600 cm^−1^ were observed. Three distinct bands of ν_3_ PO_4_ and ν_3_ HPO_4_ at 1020–1130 cm^−1^ were attributed to the typical OCP structure. The splitting of the 1022 and 1038 cm^−1^ and 1105 and 1121 cm^−1^ bands of C-OCP indicate that C-OCP has higher crystallinity than F-OCP. 

After Tris-HCl buffer immersion, the bands around 1026 and 1122 cm^−1^ of C-OCP became lower than the original C-OCP, suggesting that hydrolysis had progressed. In F-OCP, the bands around 1026 and 1037 cm^−1^ became obscure. Therefore, the hydrolysis of F-OCP was also slightly advanced.

### 2.3. Alteration of the DS in the Tris-HCl Buffer after Immersion of OCPs

The ion composition in Tris-HCl buffer incubated with OCPs was determined by chemical analysis of the supernatants at 7 days (Table 1). Nano-OCP, which consisted of nanometer-sized OCPs, was prepared to investigate whether the solubility equilibrium depends on the different crystal sizes of OCPs. The concentration of Ca^2+^ in the buffer incubated with C-OCP was higher than that of Nano-OCP and F-OCP. The inorganic phosphate (Pi) ion concentration in the buffer incubated with C-OCP was lower than in the buffer incubated with Nano-OCP and F-OCP. Although the pH values for F-OCP and C-OCP were slightly higher than those for the original buffer, the pH values for Nano-OCP were similar to the original. The calculated values of DS for calcium phosphates in the supernatants are also displayed in Table 1. The values of DS indicate that the buffers were supersaturated with respect to HA, regardless of the incubation of OCPs. The DS with respect to HA tended to increase with increasing crystal size. Furthermore, the buffer was slightly supersaturated with respect to OCP but undersaturated with respect to dicalcium phosphate dihydrate (DCPD), respectively. The buffer incubated with Nano-OCP tended to achieve saturation for OCP compared to F-OCP and C-OCP.

### 2.4. Change of Crystal Structure of OCPs in Rat Abdominal Subcutaneous Pouches

To investigate whether the crystal structure of OCP changes in vivo, we analyzed the spectroscopic characteristics of OCP by implanting F-OCP and C-OCP in rat abdominal subcutaneous pouches.

**Figure 4:**
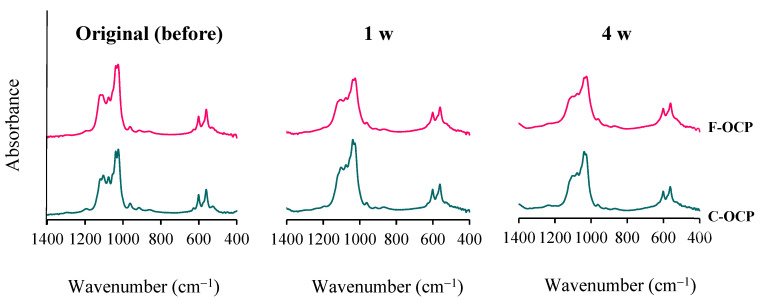
Changes in the FTIR spectra of F-OCP and C-OCP at the original (before), 1 week, and 4 weeks after implantation of rat abdominal regions.

Figure 4 shows FTIR spectra of OCPs before (original), 1, and 4 weeks after implantation in rat abdominal subcutaneous pouches. Two sharp adsorption bands originating from ν_4_ PO_4_ of around 560–600 cm^−1^ were observed in all samples. Three distinct bands of ν_3_ PO_4_ and ν_3_ HPO_4_ at 1020–1130 cm^−1^ have been attributed to the typical OCP structure and were observed in the original OCPs before implantation. In F-OCP, the band around 1026 cm^−1^ and 1038 cm^−1^ became obscure, suggesting that hydrolysis of F-OCP had slightly progressed. F-OCP maintained the three bands of original OCP until 4 weeks after the implantation. In contrast, there was a clear difference of FTIR spectra between C-OCP at 4 weeks after implantation and C-OCP original. The band around 1025 cm^−1^ and 1119 cm^−1^ of C-OCP became lower in C-OCP collected at 1 and 4 weeks after implantation, suggesting that OCP hydrolysis advances and the structure becomes more apatitic [11]. 

### 2.5. Micro-CT Analysis of Rat Tibia Defects Implanted with Various Crystal Size of OCPs

**Figure 5:**
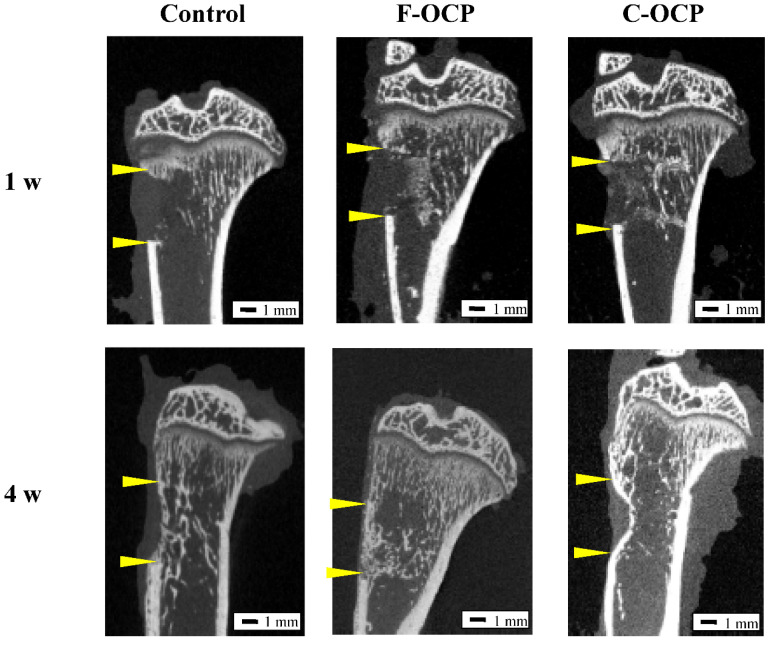
Micro-CT images of rat tibia at one and four weeks after implantation of F-OCP and C-OCP granules. Control group means bone defect without material implantation. Bars in the panels represent 1 mm. Yellow arrowheads indicate the edge of the defects.

To investigate whether the crystal microstructure of OCP affects bone formation ability, we implanted F-OCP and C-OCP in rat tibia defects. Figure 5 shows the micro-CT images of the rat tibia in sagittal plane at 1 and 4 weeks after implantation of F-OCP and C-OCP. Micro-CT images were also taken in the control group (defect only). Granular radiopaque areas corresponding to OCP crystals were observed inside the tibia defects treated with F-OCP and C-OCP at 1-week post-implantation. When F-OCP was implanted, the lineal radiopaque region corresponding to the cortical bone was connected at 4 weeks post-implantation. A similar linear radiopaque region was also observed in the C-OCP and control groups at 4 weeks post-implantation; however, it remained in the intramedullary bone. This result indicates that F-OCP promoted new bone formation in the C-OCP and control groups.

**Figure 6:**
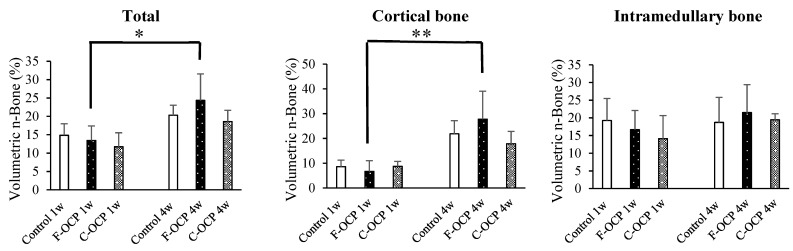
Micro-CT volumetric analysis of newly formed bone percentage of total, cortical, and intramedullary bone treated with no materials (control), F-OCP, and C-OCP at 1 week and 4 weeks post-implantation (** *p* < 0.01, * *p* < 0.05).

Figure 6 shows the volumetric percentage of newly formed bone (Volumetric n-Bone (%)) in the defect of the rat tibia after 1 and 4 weeks post-implantation. The volumetric percentage of newly formed bone in the cortical bone area was significantly higher in the F-OCP group at 4 weeks (27.8%) than in the F-OCP group at 1 week (6.7%). In the cortical bone area, new bone formation tended to advance around the F-OCP (27.8%) and inhibit around the C-OCP (17.9%) compared with control (21.9%) at 4 weeks of implantation. In the intramedullary bone area, there was no significant difference in all groups. The bone formation rate in the total area was the highest in F-OCP at 4-week post-implantation. These results suggest that F-OCP shows better cortical bone regeneration than C-OCP in rat tibia defects.

### 2.6. Histological and Histomorphometric Examinations

**Figure 7:**
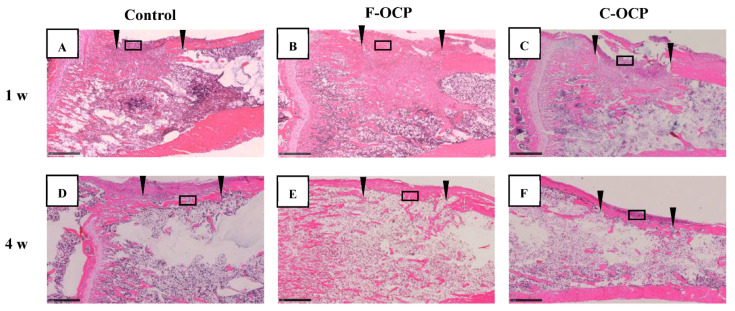
Overview around tibia defect regions of histological sections stained with hematoxylin and eosin after treatment with no materials (control) (**A**,**D**), F-OCP (**B**,**E**) and C-OCP (**C**,**F**) at 1 week (**A**–**C**) and 4 weeks (**D**–**F**) post-implantation. Scale bars in the panels represent 1 mm. The open squares indicate the areas shown as higher magnified images in Figure 8. Arrowheads indicate the edge of the defects.

**Figure 8:**
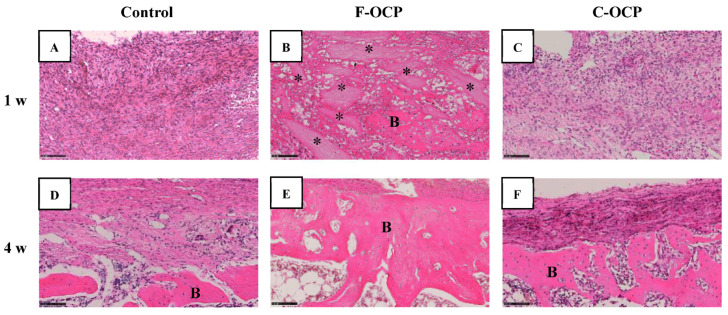
High-magnification images of tibia defect regions of histological sections stained with hematoxylin and eosin after treatment with no materials (control) (**A**,**D**), F-OCP (**B**,**E**) and C-OCP (**C**,**F**) at 1 week (**A**–**C**) and 4 weeks (**D**–**F**) post-implantation. Bars in the panels represent 100 μm. B, newly formed bone; asterisks (*), remaining OCP granules.

Figure 7 and Figure 8 show the hematoxylin and eosin (HE) staining of rat tibia defects after implantation of F-OCP or C-OCP. At the one week after implantation, new bone formation was observed in the intramedullary bone in all groups (Figure 7A–C and Figure 8A–C). In F-OCP, newly formed bone was also observed around the implanted OCP in the cortical bone region (Figure 8B). In contrast, inflammatory cell infiltration appeared around the C-OCP in the cortical bone area (Figure 8C).

Four weeks after implantation, a thick new bone was formed in the cortical bone area in the F-OCP (Figure 7E and Figure 8E). On the other hand, for the C-OCP, a layer of new bone was still observed in the intramedullary bone area and did not recover to the level of cortical bone (Figure 7F and Figure 8F). In addition, fibrous tissue remained in the cortical bone area in the C-OCP group (Figure 8F). Similarly, in the control group, new bone was observed in the cortical bone area; however, the thickness of the bone did not recover, and fibrous tissue remained (Figure 7D and Figure 8D).

**Figure 9:**
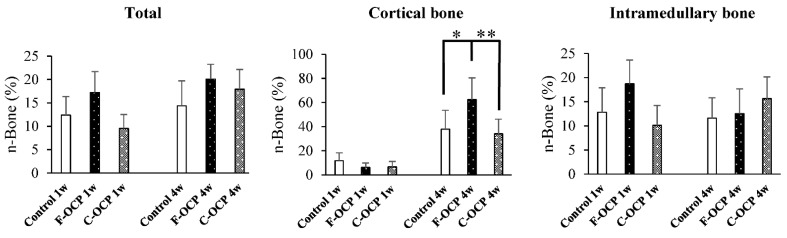
Histomorphometric analysis of newly formed bone area of total, cortical, and intramedullary bone treated with no materials (control), F-OCP, and C-OCP at 1 week and 4 weeks post-implantation (** *p* < 0.01, * *p* < 0.05).

Figure 9 shows the percentage of newly formed bone (n-Bone (%)) in the defect of the rat tibia after 1- and 4-weeks post-implantation. The percentage of newly formed bone in the cortical bone area was significantly higher in the F-OCP group (62.4%) than in the C-OCP and control groups at 4 weeks. In the intramedullary bone area, new bone formation tended to advance around the F-OCP at 1 week and decreased until 4 weeks. The bone formation rate in the total area was the highest in F-OCP at 1-week post-implantation. These results suggest that F-OCP exhibits higher bone formation ability than C-OCP in rat tibia defects. 

### 2.7. TRAP Staining

**Figure 10:**
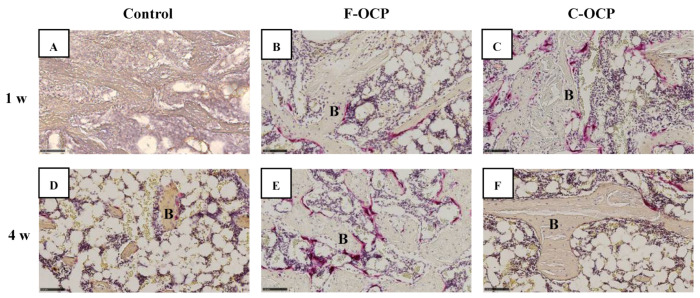
High-magnification images of tibia defect in the intramedullary bone regions of histological sections stained with TRAP after treatment with no materials (control) (**A**,**D**), F-OCP (**B**,**E**), and C-OCP (**C**,**F**) at 1 week (**A**–**C**) and 4 weeks (**D**–**F**) post-implantation. Bars in the panels represent 100 μm. B, newly formed bone.

Figure 10 shows the TRAP staining of rat tibia defects in the intramedullary bone area. Numerous TRAP-positive cells were observed around the C-OCP implantation sites at 1 week (Figure 10C). TRAP-positive cells in the F-OCP and C-OCP groups appeared around the edge of the newly formed bone (Figure 10B,C,E,F). 

**Figure 11:**
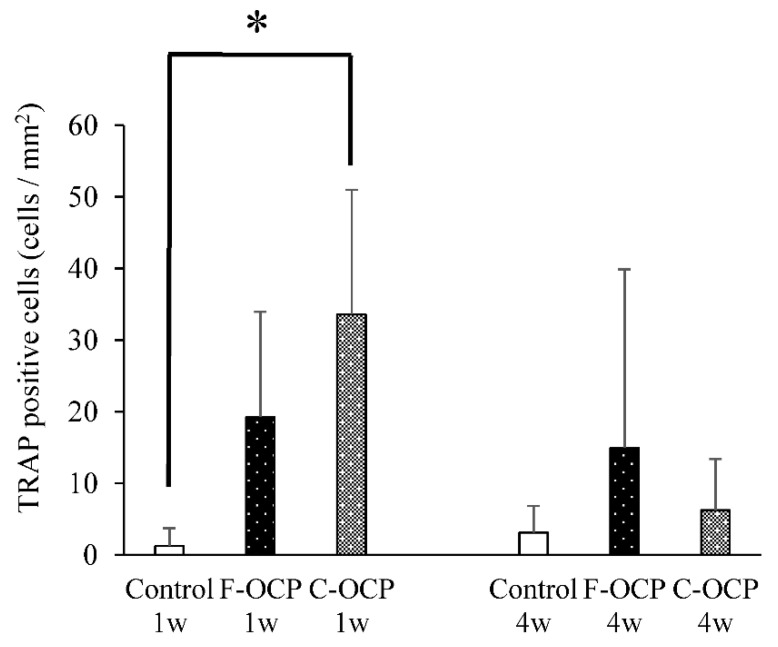
Quantitative analysis of the number of TRAP-positive cells per unit area of intramedullary bone in defects treated with no materials (control), F-OCP, and C-OCP at 1 week and 4 weeks post-implantation (* *p* < 0.05).

Figure 11 shows the quantitative analysis of TRAP-positive cells around the implantation site. At 1-week post-surgery, the number of TRAP-positive cells in the C-OCP group was significantly higher than that in the control group and tended to be higher than that in the F-OCP group. At 4 weeks after implantation, the number of TRAP-positive cells in all groups decreased over time.

## 3. Discussion

The physicochemical factors of calcium phosphates affect cellular responses and bone formation. For example, factors such as the Ca/P molar ratio [24], solubility [1], crystal phase [18], crystallinity [19], crystal size [3], crystal microstructure [25,26], porosity [27,28,29], and granule size [2] of calcium phosphates influence the cell response of osteoblasts or osteoclasts and bone formation. However, it is still unclear whether the crystal size of calcium phosphate with the same crystal phase alters osteogenesis. This study investigated the effects of different crystal sizes of OCPs on the cell response and bone formation in rat tibia defects.

Based on the results of XRD and FTIR, it was confirmed that F-OCP had lower crystallinity, and the conversion rate from OCP to HA tended to be slower (Figure 2, Figure 3 and Figure 4). In addition, F-OCP showed faster healing of cortical bone and better bone regeneration ability when implanted into rat tibia bone defects (Figure 5, Figure 6, Figure 7, Figure 8 and Figure 9). However, C-OCP tended to have high crystallinity and a fast conversion rate to apatite (Figure 2, Figure 3 and Figure 4). C-OCP implantation in the rat tibia bone defect showed many inflammatory cells in the early stage (Figure 7 and Figure 8). Numerous TRAP-positive osteoclast-like cells also appeared 1 week after implantation of C-OCP (Figure 10 and Figure 11).

This study reveals that the microstructure of OCP crystals is also one of the factors affecting bone regeneration capacity in long bones. It has been reported that the microstructure of calcium phosphates affects the function of bone-derived cells [30,31]. We have previously shown that F-OCP enhances the adhesion of mouse bone marrow-derived stromal ST2 cells and promotes bone regeneration in mouse calvarial defects compared to C-OCP [3]. The cranium is derived from neural crest tissues and is formed by membranous ossification [22]. In contrast, long tubular bones, such as the tibia, are calcified by endochondral ossification, in which cartilage is replaced by bone tissue [23]. Therefore, since the process of ossification is different between the calvaria and tibia, it is not always the case that the same biological response occurs when a bone substitute material is implanted in the defect. However, in this study, F-OCP showed a high bone regeneration capacity in tibia bone defects and calvarial defects.

The hydrolysis process from OCP to HA is also one of the factors that may affect osteoconductive and immune responses in rat bone marrow. In this study, XRD and FTIR analyses revealed that C-OCP was highly crystalline and showed a high conversion rate to HA by Tris-HCl buffer immersion and abdominal implantation (Figure 2, Figure 3 and Figure 4). In contrast, F-OCP had low crystallinity and a slow conversion rate to HA (Figure 2, Figure 3 and Figure 4). We previously reported that slightly hydrolyzed OCP (low-crystalline OCP) was found to inhibit early inflammatory reactions and osteoclastogenesis. The slightly hydrolyzed OCP promoted bone regeneration more than OCP with high crystallinity in rat tibia defects [19]. Based on the results of the previous study, it is suggested that F-OCP may promote bone regeneration due to a milder initial inflammatory response and osteoclastogenesis because its crystallinity is similar to that of partially hydrolyzed OCP.

In the immersion experiment in the Tris-HCl buffer, the Ca^2+^ concentration was lower in F-OCP than in C-OCP after 7 days of immersion (Table 1). The Pi concentration was higher in the F-OCP than in the C-OCP (Table 1). The pH of the solution was slightly lower in F-OCP than in C-OCP (Table 1). The DS of F-OCP and C-OCP were calculated under these conditions, and the DS values to OCP and HA was similar for F-OCP and C-OCP, although its value tended to somewhat decrease with decreasing the crystal dimension (Table 1). Therefore, F-OCP and C-OCP do not differ remarkably in the ease of precipitation of OCP and HA. F-OCP and C-OCP implanted in the abdomen showed a tendency to convert to HA over time; however, FTIR analysis showed that both OCPs were intermediates in the hydrolysis of OCP to HA after 4 weeks of implantation (Figure 4). These results suggest that, although the final crystal structures of F-OCP and C-OCP are similar after 4 weeks of implantation, in vivo responses (initial inflammation, osteoclastogenesis, and bone regeneration) are controlled through probable different ionic behaviors of Ca^2+^ and Pi around the material when implanted in the tibial bone defect.

Inflammatory reactions during biomaterial implantation profoundly affect the resorbability and bone regeneration capacity of the material [16,32]. It is also known that the crystal phase of calcium phosphates (OCP, β-TCP, and HA) alters osteoclastogenesis and crosstalk between osteoclasts and osteoblasts [18]. In the present study, inflammatory cell infiltration was observed in C-OCP 1 week after implantation (Figure 7 and Figure 8). In addition, the number of TRAP-positive cells after 1 week of implantation was higher than that in the control group (Figure 10 and Figure 11), suggesting that the formation of osteoclast-like cells actively occurred in the initial stage of implantation. In contrast, in F-OCP, very few inflammatory cells were observed 1 week after implantation (Figure 7 and Figure 8). The percentage of newly formed bone in the total area tended to be higher in the F-OCP group than that in the control and C-OCP groups (Figure 9). These results suggest that bone regeneration was delayed in C-OCP due to the early inflammatory reaction and accelerated osteoclast formation, whereas in F-OCP, the inflammatory reaction may be subsided at a very early stage, and the bone regeneration phase is rapidly initiated.

## 4. Materials and Methods

### 4.1. Preparation of Various Crystal Sizes of OCPs

Fine-OCP (F-OCP) and coarse-OCP (C-OCP) were synthesized and controlled using a wet method [3,33]. F-OCP and C-OCP granules with particle sizes of 300–500 μm were prepared by sieving the material between 32 and 48 mesh. To prepare for Nano-OCP, 100 mg of F-OCP was pulverized using a multi-bead shocker (Yasui Kikai Co., Osaka, Japan) at 2500 rpm for 30 s [34] and washed by ultrapure water. After lyophilization, Nano-OCP granules were also sieved between 300 to 500 μm.

The crystal microstructures of each granule were analyzed by field emission scanning electron microscopy (FE-SEM, JSM-7100F, JEOL, Tokyo, Japan) operated at an accelerating voltage of 5.0 kV. Carbon sputtering was performed before observation.

### 4.2. Characterization of OCPs and Supernatants after the Immersion in the Tris-HCl Buffer

Five milligrams of F-OCP, C-OCP, and Nano-OCP granules were immersed in 1 mL of Tris-HCl buffer solution (150 mM, pH = 7.4) for 7 days. These materials were washed with water several times and lyophilized. The crystal structures of the original OCPs and those collected on day 7 after immersion in the Tris-HCl buffer were examined by X-ray diffraction (XRD) and Fourier transform infrared spectroscopy (FTIR). The XRD pattern was recorded using step scanning at 0.02° intervals from 3.0° to 60°, with CuKα radiation on a diffractometer (MiniFlex 300; Rigaku Co., Ltd., Tokyo, Japan) at 40 kV and 15 mA.

### 4.3. Implantation of OCPs into Rat Tibia Defects or Subcutaneous Tissue

In this study, 12-week-old male Wistar rats were used for animal experiments. We followed the principles of standard laboratory animal care and national laws. All protocols for animal handling and treatment were approved by the Animal Research Committee of Tohoku University (approval number 2018DnA-040).

The implantation of OCPs into rat tibia defects followed previously reported procedures [16,20]. After inhalation of isoflurane, the rats were anesthetized with three types of mixed anesthetic agents (5 μL/g) consisting of medetomidine (7.5 μg/mL), midazolam (0.4 mg/mL), and butorphanol (0.5 mg/mL). The skin and periosteum of the tibia were dissected and raised from the bone surface. A bone defect (3.0 mm in diameter, 2.5 mm in depth) was made in the medial cortex of the right tibia of a rat using a fissure bur under continuous irrigation with sterile saline. The bone cavity was washed with sterile saline to remove debris completely. The cavity was filled with OCPs (4.1 mg), and the wound was closed. In the control group, animals received bone defects without material implantation.

To investigate the effect of crystal size on the physicochemical changes of OCP in vivo, each OCP was placed on the abdominal subcutaneous tissues of rats. Subcutaneous incisions were made vertically and horizontally along the midline of the rat’s abdomen. OCP was implanted in four places on the abdominal subcutaneous tissues at 20 mg each (80 mg in total), and the incision was sutured.

The animals were sacrificed with an overdose of anesthesia, and the samples were collected at 1 and 4 weeks post-implantation for fixation and analysis.

### 4.4. Characterization of OCPs Collected after Implantation of Rat Abdominal Regions

OCPs collected from rat abdominal areas were washed and lyophilized before analysis. The spectra of the materials were obtained by a Fourier transform infrared spectroscope (FTIR) (FT/IR-6300, JASCO, Tokyo, Japan) with the sample diluted in KBr over a range of 4000–400 cm^−1^ with 4 cm^−1^ resolution.

Ca^2+^ and inorganic Pi ion concentrations in the Tris-HCl buffer at day 7 were determined quantitatively using calcium E tests and Phosphor C tests (Wako Pure Chemical Industries, Osaka, Japan), respectively. The pH of the solution was measured using a pH electrode (9618S-10D, HORIBA, Ltd., Kyoto, Japan).

### 4.5. Determination of the DS in the Tris-HCl Buffer Immersed with F-OCP, C-OCP and Nano-OCP

The DS collected in the Tris-HCl buffer immersed in OCPs was calculated to estimate the solubility with respect to HA, OCP, and DCPD in the media. The DS can be expressed by dividing the ionic product by the solubility product of the objective calcium phosphate phases. DS values equal to 1.0, <1.0, and >1.0 correspond to the conditions of saturation, undersaturation, and supersaturation, respectively. The ionic activity products for calcium phosphate are usually calculated using the analytical results of [Ca], [Mg], [Na], [K], [Pi], [Cl], and [F], as well as the pH value, in conjunction with the three mass balance equations for [Ca], [Pi], and [Mg], according to previous reports [35,36,37]. In the present study, the pH and concentration of Ca^2+^ and Pi obtained using chemical analyses were used. The ion pairs considered were CaH_2_PO_4_^+^, CaHPO_4_^0^, MgHPO_4_^0^, CaHCO_3_^+^, and MgHCO_3_^+^. The DS was estimated in terms of the mean ionic activity products with respect to HA, OCP, and DCPD. Furthermore, 150 mM background electrolyte as Na^+^ was used as an ionic strength. Mg^2+^ and F^−^ are assumed to be approximately zero. The solubility product constants used were 7.36 × 10^−60^ (mol/L)^9^ for HA [38], 2.51 × 10^−49^ (mol/L)^8^ for OCP [39], and 2.77 × 10^−7^ (mol/L)^2^ for DCPD [40] at 37 °C.

### 4.6. Micro-CT Analysis

Tibias after implantation of various crystal sizes of OCPs were examined with a microfocus X-ray computed tomography system (micro-CT; Scan Xmate-E090, Comscantecno Co., Ltd., Kanagawa, Japan) operating at 90 kV and 100 μA. The samples were rotated 360°. Image resolution was fixed at a pixel size of 12.902 μm. The magnification was 3.875×. Each 3D image dataset consisted of approximately 600 micro-CT slide images (1024 × 1008 pixels) with 16-bit-gray levels. The voxel size was 50 × 50 × 50 μm^3^.

For volumetric analysis, the percentage of newly formed bone [*Volumetric n* − *Bone* (%)] in the cortical or intramedullary bone regions was calculated using Equation (1):

(1) $$Volumetric\;n-Bone\;\left(\%\right)=\frac{V\_n-Bone\;\left(mm^{3}\right)}{V\_tot\;\left(mm^{3}\right)}\times 100$$

where {*V_n* − *Bone*} (mm^3^) is the newly formed bone volume and {*V_tot*} (mm^3^) is the total, cortical, or intramedullary bone volume. The volume of cortical bone was determined using the thickness of the original cortical bone as an index. The overall volume was determined to be the largest cylindrical shape that would not include the opposite cortical bone. The volume of the intramedullary volume was obtained by subtracting the volume of the cortical bone from the overall volume.

### 4.7. Tissue Preparation and Histomorphometric Analysis

The tissue was fixed with 10% formalin solution at each time point after implantation and decalcified in 10% ethylenediaminetetraacetic acid (EDTA). The samples were dehydrated in a graded series of ethanol and embedded in paraffin using a cell and tissue processor (CT-Pro20, GenoStaff, Tokyo, Japan). Sections were cut to 4 μm thickness and stained with hematoxylin and eosin (HE).

**Figure 12:**
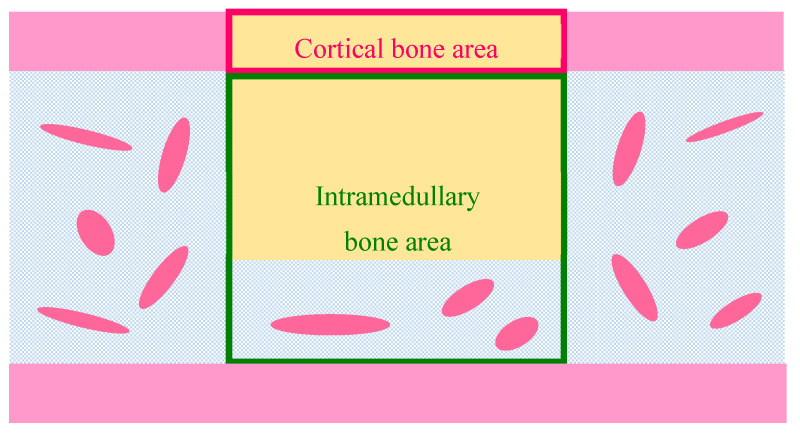
Schematic illustration of histomorphomeric measurement of newly formed bone [*n* − *Bone* (%)] in the defect. Pink frame, cortical bone area; yellow rectangle, bone defect; green frame, intramedullary bone area.

For histomorphometric analysis, the percentage of newly formed bone [*n* − *Bone* (%)] in the cortical or intramedullary bone regions was calculated using Equation (2):

(2) $$n-Bone\;\left(\%\right)=\frac{A\_n-Bone\;\left(mm^{2}\right)}{A\_tot\;\left(mm^{2}\right)}\times 100$$

where {*A_n* − *Bone*} (mm^2^) is the newly formed bone area and {*A_tot*} (mm^2^) is the total, cortical, or intramedullary bone area. The cortical bone area was determined by the average cortical bone width estimated from the thickness of the proximal and distal cortical bone sections. The intramedullary bone area was defined as the entire area minus the cortical bone area on the contralateral and defect sides (Figure 12).

### 4.8. Tartrate-Resistant Acid Phosphatase (TRAP) Staining

Tartrate-resistant acid phosphatase (TRAP) and cell nuclei were stained after deparaffinization. TRAP solution was prepared by mixing 40 mM sodium acetate, 50 mM sodium tartrate, naphthol AS-MX phosphate, and N, N-dimethyl formamide (all obtained from Sigma-Aldrich Co. LLC, Saint Louis, MO, USA), and the pH was adjusted to 5.0. The sections were stained with Fast Red Violet LB (Sigma-Aldrich Co. LLC, Saint Louis, MO, USA) dissolved in TRAP solution for 1 h at 37 °C. The samples were counterstained with hematoxylin. The number of multinucleated TRAP-positive cells with purplish red colors per unit area of cancellous bone was counted in the photographs of the section, acquired using a virtual slide scanner at 200× magnification.

### 4.9. Imaging Techniques

Photographs of HE and TRAP staining were taken using a virtual slide scanner (NanoZoomer^®^, Hamamatsu Pho-tonics K.K., Hamamatsu, Japan) at 25× and 200× magnification. The pixel size of each picture was adjusted to 1920 × 1128 (pixels) and analyzed using NDP. View 2 software (Hamamatsu Pho-tonics K.K., Hamamatsu, Japan).

Micro-CT volumetric analysis was performed using OsiriX MD software (Pixmeo, Switzerland).

For histomorphometric analysis, the area of newly formed bone and total was selected using Adobe Photoshop CS 5.1. Each region of the photograph was quantified using the ImageJ software (National Institutes of Health, Bethesda, MD, USA).

### 4.10. Statistical Analysis

Results are expressed as mean ± standard deviation (SD). Statistical differences among specimens were evaluated by Tukey–Kramer multiple comparison analysis using Statcel 4 software (OMS Publishing Inc., Saitama, Japan). Statistical significance was set at *p* < 0.05.

## 5. Conclusions

This study revealed that F-OCP with smaller crystal lengths promoted rapid bone regeneration, especially in the cortical bone. In contrast, infiltration of inflammatory cells and promotion of osteoclast formation were observed around C-OCP with larger crystal lengths. The microstructure of OCP crystals affects the ionic environment in vitro. The concentration of Ca^2+^ in Tris-HCl buffer incubated with C-OCP was higher than that of F-OCP. The Pi ion concentration in the buffer incubated with C-OCP was lower than that of F-OCP. These differences between F-OCP and C-OCP were related to the conversion process from OCP to HA. Therefore, the microstructure of OCP crystals also influences ionic environment possibly in vivo, alters their cellular responses, and significantly changes the bone regeneration ability of rat tibia defects. Further analysis will be necessary to investigate the effect of the microstructure of OCPs on the differentiation and function of bone-related cells.

## Figures and Tables

**Table 1:** Degree of supersaturation (DS) of Tris-HCl buffer (pH = 7.4) incubated with OCPs.

	Periods (day)	Calcium (mM)	Phosphate (mM)	pH	DS at pH (Each) and 37 °C		
					HA	OCP	DCPD
control	7	0.056	0.010	7.470	Not calc.	Not calc.	Not calc.
Nano-OCP	7	0.331	1.610	7.475	7.781 × 10^8^	9.406 × 10	1.763 × 10^−1^
F-OCP	7	0.363	1.576	7.486	1.298 × 10^9^	1.359 × 10^1^	1.900 × 10^−1^
C-OCP	7	0.642	0.669	7.522	2.807 × 10^9^	1.367 × 10^1^	1.475 × 10^−1^

## Data Availability

The data were basically provided in the manuscript.
